# Improving the uptake of pre-travel health advice amongst migrant Australians: exploring the attitudes of primary care providers and migrant community groups

**DOI:** 10.1186/s12879-016-1479-1

**Published:** 2016-05-18

**Authors:** Holly Seale, Rajneesh Kaur, Abela Mahimbo, C. Raina MacIntyre, Nicholas Zwar, Mitchell Smith, Heather Worth, Anita E Heywood

**Affiliations:** School of Public Health and Community Medicine, UNSW Medicine, University of New South Wales, Sydney, Australia; National Centre for Immunisation Research and Surveillance, Children’s Hospital at Westmead, Sydney, Australia; NSW Refugee Health Service, Sydney, Australia

**Keywords:** Vaccination, Travel, Migrants, Attitudes, Visiting friends and relatives, Infectious diseases

## Abstract

**Background:**

Migrant travellers who return to their country of origin to visit family and friends (VFR) are less likely to seek travel-related medical care and are less likely to adhere to recommended medications and travel precautions. Through this study, we aimed to get an understanding of the views of stakeholders from community migrant centres and primary care providers on barriers for migrants, particularly from non-English speaking backgrounds, in accessing travel health advice and the strategies that could be used to engage them.

**Methods:**

A qualitative study involving 20 semi-structured interviews was undertaken in Sydney, Australia between January 2013 and September 2014. Thematic analysis was undertaken.

**Results:**

Language barriers, a lower perceived risk of travel-related infections and the financial costs of seeking pre-travel health care were nominated as being the key barriers impacting on the uptake of pre-travel health advice and precautions. To overcome pre-existing language barriers, participants advocated for the use of bilingual community educators, community radio, ethnic newspapers and posters in the dissemination of pre-travel health information.

**Conclusions:**

Travel is a major vector of importation of infectious diseases into Australia, and VFR travellers are at high risk of infection. Collaboration between the Government, primary care physicians, migrant community groups and migrants themselves is crucial if we are to be successful in reducing travel-related risks among this subgroup of travellers.

## Background

Migrants who travel to their country of origin to visit friends and relatives (VFR) are considered to be at higher risk of contracting travel-related illness when compared to other groups of travellers [1–3]. The “classic” definition for a VFR traveller includes: ethnicity of the traveller which is different to the host country population but which is similar to the ethnicity of their destination, intended purpose of travel to visit family members or friends and the intended destination having a higher level of risk for specific diseases such as tropical infectious diseases [4].

VFR travellers are more likely to travel for longer periods of time, to travel to rural destinations and may make multiple return visits [5]. They often stay with family members or friends, have less control over their diets and are more likely to drink untreated water [6–8]. Previous studies have described VFR travellers at being at increased risk of malaria, viral hepatitis, HIV/AIDs in comparison to tourists and business travellers [9, 10].

Barriers to the delivery of pre-travel medical services for this sub-group of travellers exist at many levels, including at the: (1) systems level (access to services), (2) patient level (misperception of disease risk), and (3) provider level (lack of resources, inadequate training in travel medicine and misperception of risk). Previous studies have established that VFR travellers perceive less personal risk or threat when travelling to their country of origin, stemming from a sense of familiarity with the destination country and its infectious disease risks. Cultural beliefs and language barriers are also important factors associated with suboptimal uptake of pre-travel advice among VFR travellers [11]. Lastly, not only do the actual financial costs (costs of visiting a healthcare provider and vaccines) impact negatively on the uptake of pre-travel health advice, but also the negative perceptions of travellers towards the cost-benefits of pre-travel preparation. It has also been suggested that when VFRs seek pre-travel advice, they often visit clinicians sharing similar ethnicities and beliefs who then influence their perception on pre-travel prevention strategies [12]. Primary care physicians may have competing priorities when treating patients, may not ask about upcoming trips or may assume that migrants have previously received travel-related health services because of the frequent repeated trips they may make to their country of origin. Other factors that may hinder the uptake of pre-travel advice are the inadequacies of travel medicine guidelines and published material.

In order to engage VFR travellers and improve the uptake of pre-travel health advice, appropriate vaccination and the use of other travel health precautions, it has been suggested that innovative communication strategies are required [9, 13, 14]. To date, there have been a number of attempts to implement community-based initiatives to engage VFR travellers, however very few have documented the outcomes of these programs. In order to develop a strategy to improve the uptake of travel health advice and vaccination amongst VFR travellers, our team felt it was important to firstly get a better understanding of the barriers impacting on the delivery of health advise, with a focus on travel health, to migrants and the strategies that could be used to overcome these.

## Methods

### Study design

In-depth semi-structured interviews were undertaken with stakeholders located in Sydney, Australia. Ethics approval was obtained from the Human Research Ethics Advisory (HREA) Panel of the University of New South Wales, Sydney Australia (Ref No 2012-7-26).

### Participants and recruitment

Stakeholders were defined as ‘any person, group, or organisation that is affected by the causes or consequences of an issue’ [15]. We recruited staff members from Migrant Resource Centres (MRCs). These community non-profit centres provide a diverse range of assistance and care services to people of culturally and linguistically diverse (CALD) backgrounds. These centres are often staffed by migrants from similar backgrounds/experiences to the community groups they assist and are experienced at reaching out to newly arrived migrants in the community. While these groups are not focused on providing travel related advice, they have experience with delivering other health messages to hard to reach groups. In addition, we reached out to Chinese and Indian (two of the largest groups of VFR travellers) non-government community organisations based in Sydney. We also recruited primary care physicians practising in areas of Sydney with high migrant populations.

Participants from community organisations/MRCs were recruited to the study via a number of approaches. First, an online search of websites was conducted to identify potential eligible candidates. Each candidate was then followed up via email with an invitation letter. Second, interested candidates were asked to recommend any colleagues who would be willing to participate as well. We targeted primary care physicians practising in areas of Sydney, New South Wales (NSW), with high migrant populations. A list of primary care physicians was compiled based on publically available information from online medical directories. In addition, primary care physicians from CALD backgrounds who had previously taken part in research projects were approached. Participants were only included into the study when full written consent had been received. This study did not collect any identifiable personal information from the participants. A shopping voucher was given to all participants to compensate them for their time.

### Data collection

An interview guide was jointly developed and reviewed by the researchers to identify key areas of interest for the study. This included a series of questions related to the following topics: perceptions about travel-related risks for VFRs and other travellers, barriers to accessing pre-travel health information/advice and suggestions on the most effective strategies or modes of delivery of pre-travel health information that will enhance the uptake of pre-travel health information/advice among VFRs in Australia. The list of topics served only as a general direction during each interview with the interviewers asking questions in an open-ended manner to allow room for expansion and using paraphrasing and additional questions to seek clarification. This ensured that the study included most of the topics and was flexible to changes depending on the actual scenario. Prompts were only given when the interviewer deemed they were required to encourage the conversation back to topic. HS and RK conducted all interviews via telephone. Participating community representatives were encouraged to narrate events and situations they had experienced and to describe the circumstances around their behaviours and choices. The stakeholder’s actual experiences were the focus, rather than their opinions.

### Analysis

All interviews were audio recorded, transcribed verbatim and analysed thematically. Transcripts for each taped interview was checked for internal consistency and corroborated with other interviews. The researchers used NVivo 10 for data management. Following repeated and close reading of the individual interview transcripts, three researchers (HS, RK and AM) independently constructed a code list of major themes emerging from the data. These code lists were compared and crosschecked and a final list was compiled. An agreed thematic framework was then applied to another subsample of transcripts and further modified. Using this final framework, the remaining transcripts were analysed and coded.

## Results

Twenty stakeholders consented to participate and were interviewed (8 staff members from MRCs, 2 community representatives, and 10 primary care providers) between January 2013 and September 2014.

### Misconceptions about risk and level of protection

There was a perception among the participants from the MRC and community groups that non-VFR travellers were at a higher risk of acquiring travel-related illnesses compared to VFRs, because the latter group were likely to be immune to acquiring communicable diseases because they are just “going home”. It was suggested that the higher travel-related risks posited of non-VFRs could be explained by a tendency to be more adventurous and a lack of familiarity with the destination and “appropriate coping mechanisms” thus predisposing them to higher risks.

*“But it’s not that they’re going from here. So a lot of people, who are born and bred there, adult migrants, I don’t think there is any reason for them to have any injections because their body is still resilient to, you know, the upbringing there.” (MRC Staff member)*

Community representatives did acknowledge that although they perceived the risk of travel-related illnesses was higher among non-VFR travellers than VFRs, the risk of infection could be particularly heightened among VFRs who visit rural/remote areas. Behaviours such as eating from street food vendors and extended stays in rural and remote areas without the use of any prophylaxis were perceived to be associated with heightened risk.

Primary care physicians felt that migrants had misconceptions about their risk based on the notion that they had lived in the community, therefore must be immune to the diseases in their country of origin. In addition, one primary care physician suggested that migrants generally believe that they have had all their childhood vaccination and are thus immune to other diseases as well. It was suggested that these misconceptions contribute to the low level of pre-travel health seeking.

*“I think the situation they have lived in and what they have suffered through, they probably have a better understanding than non-migrants … they’re more resistant than the non-migrants.” (MRC Staff member)*

*“Typically, I will be the person to bring up about travel risks. I think, from that person’s perspective, they’ve sort of lived there for a very substantial part of their lives, so they weren’t specifically thinking that … it’s a sense of going back to, going back home, going back to, a home context. As opposed to necessarily going to anywhere exotic or unusual.” (Primary care physician)*

It was postulated that the level of awareness towards travel-related risks was not only associated with the length of time that they had lived in Australia for but it was also based on whether they had integrated into Australian culture.

*“Like, if they have lived here for quite a long time, like 20–25 years, and they have got friends who are Australians and they are quite integrated in the community, then the level of awareness is greater.” (Primary care physician)*

Alternatively, one primary care physician suggested that patients of older age are more resistant to advice. Participants were of the opinion that VFR travellers are well aware of the risks they might experience in their home country and are generally aware of the diseases endemic in their country. A few of the primary care physicians thought that migrants are aware of general health issues such as enteric infections and influenza, so observe general precautions, but are not very aware of vaccine preventable diseases. It was suggested by both MRC staff and primary care physicians that even if migrants are aware that being a VFR traveller is associated with risks, it may not necessarily translate into health seeking behaviour.

*“Well you know, even if they’re aware, but they live there, so they don’t put the importance we put.” (MRC Staff member)*

*“They, they can’t see the point, they can’t see the logic in being immunised against something when they lived there for a long period of time and they weren’t immunised against those things when they lived there …” (Primary care physician)*

*“I mean, they are more resistant to the fact that they need the vaccinations, only because they have preconceived ideas that they will probably be immune because they have lived there for X number of years, or they lived there for 20 years, or they were born there in the past.” (Primary care physician)*

Participants also acknowledged the impact that rumours have in distorting information pertinent to travel health among VFRs, particularly within various migrant communities. Community representatives and primary care physicians considered it important to address the issue of myths around side effects of vaccines, as well as educating people about the importance of getting vaccinated.

### Talking about travel health

Most primary care physicians were of the view that the issue of travel medicine is ‘not brought up’ and ‘not volunteered’ by patients. When relating their experiences of communicating with migrant patients about travel health issues, primary care physicians described low levels of awareness of the risks associated with travel amongst their patients. They described patients being shocked, surprised, or amused, and others who immediately dismiss travel health information.

*“Despite having provided information, they’ve already made up their mind they’re not going to get vaccines… They’re still happy to talk about it.” (Primary care physician)*

When migrants have preconceived notions about their risk, primary care physicians expressed difficulties in providing unsolicited pre-travel health advice, as suggested in the comment below.

*“I have no problems with that. But sometimes I find it not easy … uneasy pushing this issue, particularly when the patient came in for one other reason …” (Primary care physician)*

### The cost of healthcare and precautions is the primary barrier

Participants were divided on whether there were barriers to accessibility of pre-travel health information among VFRs. Some suggested that there are no barriers to accessing pre-travel information because there are a range of publications and multiple freely available websites.

*“…The information is accessible, definitely. What I usually do when people are going back to their country, we recommend them to use the Smart Travel website, and the information is there. The information is in different languages as well.” (MRC Staff member)*

Amongst those who acknowledged barriers to access of pre-travel health information and advice, the high costs of a consultation and/or the travel precautions (such as vaccine cost) were cited as the major obstacle. Primary care physicians and community representatives felt that VFR travellers to resource poor countries may not have the financial capacity to include vaccines in their travel budget.

*“The cost of vaccines is a real issue….they’re trying to put together an amount to go and visit their dear one; and some of them, they only have the ticket money and then they live on the family there with very little money.” (MRC Staff member)*

*“If you’re going to a country where you need more than one vaccine, you’re up for a couple of hundred dollars already. At that point they probably weigh up the risk of catching something versus the costs, thinking “Nah, don’t worry about it, I’ve been there ten times before and I haven’t been sick. Why should I pay a couple of hundred bucks this time?” And the more they’ve been overseas and been back to their home country on repeated trips, the more invincible they feel.” (Primary care physician)*

Most community representatives thought that travel-related health advice is the responsibility of primary care physicians, but felt that travel health was not a priority due to lack of consultation time and competing priorities. A few primary care physicians did acknowledge that travel consultations take longer and hence may be neglected.

“*So*, *it depends on the primary care physician*, *how much work they usually do in informing people. But usually they have no time at all* – *they go in*, *go out*, *you know*, *preliminary check*, *and you know*, *sign a Medicare and off you go*, *unfortunately. You see what I mean*? *They don*’*t have the time to do* … *But for me*, *the fact that they don*’*t have a great deal of time*, *and they expect the client to take the responsibility themselves. I don*’*t think they see that as their responsibility.*” (*MRC Staff member*)

### Language as a barrier

Participants were divided on whether language was a barrier to seeking pre-travel health advice. While some acknowledged that it would be challenging for some migrants to obtain and understand or ‘unpack’ the travel health information if it is only available in English, others disagreed.

“… *Our greatest barrier is language and that*’*s not just with office workers* [*at the MRC*] *talking to migrants*, *it*’*s* … *we*’*re always having to* … *even when there*’*s written material*, *by the way*, *and translated material*, *it*’*s* … *we*’*re still having to really unpack what it means.*” (*MRC Staff member*)

However, other participants argued that language difficulties might not actually be a barrier, since travellers can opt to seek services from primary care physicians of similar ethnic backgrounds. They also stated that patients from culturally & linguistically diverse backgrounds tend to seek out primary care physicians who speak their own language.

*“If they are Chinese and they can’t speak English, definitely there would be barriers if they’re with a non-Chinese-speaking primary care physician. But I realise that most people, they will actually seek a Chinese primary care physician. So in term of that, there should not be a barrier unless they are, like you say, a very, very new migrant and they might not be able to know where to go.” (MRC Staff member)*

*“There are a lot of primary care physicians in the local area who speak many languages, so I could well imagine that, if you don’t speak English very well, that you would seek out a local primary care physician who can speak, who would speak your language and perhaps mostly get, get most of your care from that individual.” (Primary care physician)*

### Resources need to be culturally and linguistically appropriate and delivered by peers

To overcome pre-existing language barriers, participants advocated for the use of bilingual community educators, ethnic newspapers and community radio and posters in the dissemination of pre-travel health information. Holding workshops that aim to educate the community was proposed as a strategy to rectify the misconceptions around pre-travel risks and improve the uptake of pre-travel health advice and vaccination. Places of worship, migrant organisations and travel agents were also highlighted as important targets for the dissemination of information. However, several of the community representatives considered primary care physicians to be the best source of such information.

*“Whenever they’ve got information in their first language, that makes a dramatic difference because they’re able to … and in communities, and say there is a lack of literacy, then if you are literate in that community you can pass on the information, so it can go around. Whenever there’s good information in people’s own language, I think things are, the risk is much lower.” (MRC Staff member)*

Overwhelmingly, it was felt that an integrated multidisciplinary approach involving primary care physicians, health department officials, non -governmental organizations, MRC and community based organizations was needed to maximise the impact of a promotion campaign. With regards to the content of the messages, one participant suggested focusing on the health and welfare of the children, as that would capture the attention of migrants. Personal stories were considered to be more powerful and productive than other sources of information. It was emphasised that there is a lack of understanding about the ‘preferences and networks’ of different migrant groups.

*“..The message really has to come from migrants themselves….people speaking in their own language with their own experiences and explaining why, even with real scenarios.” (MRC Staff member)*

*“…If an Indian person.. is fronting a radio ad campaign saying “I was born in India, I came to Australia in 1989, I’ve been home 20 times, and the last time [I went home] I caught cholera.” (Primary care physician)*

*“And I think the best delivery is actually through community based culturally appropriate groups. I think the information is better received coming from members of their own cultural group than coming from, from a Western trained doctor who they may not perceive as understanding their own country.” (Primary care physician)*

## Discussion

We interviewed a range of stakeholders from Sydney, Australia to obtain their opinions about travel-related health risks, barriers to uptake of pre-travel information and strategies to improve pre-travel health information among VFR travellers. Compared to previous studies, our work focused on the community stakeholders and primary care providers who play a role in delivering education and health programs to migrants or co-ordinating community events. Importantly, many of our stakeholders were able to speak personally of their experiences, as they themselves were migrants to Australia.

In line with the key issues reported by migrants themselves [5], a lower perceived risk of travel-related infections, language barriers and the financial costs of seeking pre-travel health care were nominated as being the key issues by stakeholders. However, Baggett et al. suggested that while the financial costs may be a barrier for some communities, it should not be generalised that this is an issue for all migrant groups [16]. In their study of US residents traveling to India, more than 90 % of VFRs travellers had at least a college education and only 6 % cited financial barriers or lack of insurance as reasons for not seeking pre-travel health services, compared to 12 % of other travellers. Strategies may therefore need to be tailored for VFR groups of different economic means.

The literature suggests that there is a direct impact of adherence to pre-travel advice on better outcomes for travellers, if the consultation process is brief and concise, and coupled with effective two-way communication [17]. To improve the quality and appropriateness of the pre-travel consultation, primary care physicians and other healthcare professionals need to undergo specific training in the area of travel health [18].

In case of constrained finances, it is suggested that health care providers prioritize recommended pre-travel vaccines and opt for cheaper alternatives where available [1]. Routine immunization records should be reviewed for adults, as well as for their children, during their visit to a primary care provider. In addition, migrants need to be reminded that immunizations should be considered an investment toward the ‘potential traveller’s future health’, since most need not be repeated. Most importantly, information (written and verbal) regarding travel health needs to be offered in languages that are appropriate to the intending traveller [19]. Where language difficulties exist, the use of professional interpreters should be encouraged [20].

While primary care physicians are considered to be primary choice for the delivery of advice, previous studies have consistently shown that VFR travellers are less likely than other travellers to seek pre-travel health advice, particularly from a medical practitioner [5]. Given this finding, alternative settings to deliver quality travel health information are necessary. MRCs and other migrant community-led organisations have an important role in the Australian community and may be avenues for the delivery of travel health information. Many migrant organisations have established roles in connecting migrants with the health services, improving health system literacy and providing information pertinent to settlement. Building stronger partnerships between MRCs and other community led organisations and primary care physicians will assist with moving forward with this initiative. In addition, training will be required for staff at the MRCs as they are not medically trained and will need guidance about the risks associated with travel and the potential need for travel health precautions.

The use of mass media such as ethnic newspapers and radio and posters were also suggested as approaches that could be used to increase awareness of travel-related health risks. However, health promotion strategies that aim to increase awareness amongst the target groups are only effective when there is a strong collaboration and coordination between the Government, potential stakeholders from the health sector such as primary care physicians and the migrant communities [21]. Other approaches suggested by our participants included the distribution of information via places of worship and via travel agencies. The efficacy of current and alternative approaches to travel medicine counselling should be studied across VFR traveller populations.

In 2010, a small community education campaign in Victoria, Australia aimed to increase awareness regarding the need for pre-travel visits amongst VFR travellers [22]. The campaign consisted of the involvement of doctors from the targeted ethnic groups to act as spokespeople; media outreach (media releases, newspaper opinion articles and scripted Q&A for radio interviews); development of multilingual printed materials (posters, flyers and tear-sheets); and the hosting of information stalls aimed at conveying travel-related public health messages at ethnic community events. This program also worked with a communications consultancy firm and a multicultural media group to develop the initiatives. While this novel program was not formally evaluated, the authors did note that at each of the community events large crowds visited the stalls and over 5000 cards were distributed during the four festivals [22].

Research focused on the attitudes of primary care providers and community stakeholders towards VFR traveller health is relatively limited. This is one of the main strengths of the study. In addition, the use of in-depth interviews to elicit a greater depth in the information is also a key strength of our work. A limitation of this study is that interviews were only undertaken with a select group of participants (representing only some migrant groups), so the possibility of other important themes emerging cannot be ruled out. In addition, we did not collect any specific details about the participant’s occupation. This was a small, qualitative study, and the findings can inform a larger, quantitative study.

## Conclusions

VFR travellers are at increased risk of preventable infectious diseases during travel and subsequent importation of these diseases into Australia upon their return, particularly from countries in which infectious diseases are poorly controlled. With a quarter of Australia’s population being foreign-born, and a quarter of departing Australians travelling to visit friends and relatives, VFR travel is an important risk to national disease control. The diversity of ethnicities among migrants in Australia poses a challenge in addressing context specific travel-related risks among VFRs [22]. Although efforts have been made to generally increase the uptake of pre-travel health information and recommendations amongst travellers, challenges remain in regards to this sub-group. Collaboration between the Government, primary care physicians, MRCs and migrant communities themselves is crucial if we are to be successful in reducing travel-related risks among this subgroup of travellers.

### Ethics approval and consent to participate

Ethics approval was obtained from the Human Research Ethics Advisory (HREA) Panel of the University of New South Wales, Sydney Australia (Ref No 2012-7-26). Written consent was obtained from all of the participants.

### Availability of data and materials

Data and materials are available from the author upon request.

## Abbreviations

CALD: culturally and linguistically diverse
MRC: migrant resource centres
NSW: New South Wales
VFR: visiting family and friends
